# Fatp1 Deficiency Affects Retinal Light Response and Dark Adaptation, and Induces Age-Related Alterations

**DOI:** 10.1371/journal.pone.0050231

**Published:** 2012-11-16

**Authors:** Karim Chekroud, Laurent Guillou, Stephane Grégoire, Gilles Ducharme, Emilie Brun, Chantal Cazevieille, Lionel Bretillon, Christian P. Hamel, Philippe Brabet, Marie O. Pequignot

**Affiliations:** 1 Inserm U1051, Institute for Neurosciences of Montpellier, CHU St Eloi, Montpellier, France; 2 INRA, Eye and Nutrition Research Group, Dijon, France; 3 CNRS UMR5149, Institut de Mathématiques et de Modélisation de Montpellier, France; 4 CRIC/IURC 641, Montpellier, France; Children's Hospital Boston, United States of America

## Abstract

FATP1 is involved in lipid transport into cells and in intracellular lipid metabolism. We showed previously that this protein interacts with and inhibits the limiting-step isomerase of the visual cycle RPE65. Here, we aimed to analyze the effect of *Fatp1*-deficiency *in vivo* on the visual cycle, structure and function, and on retinal aging. Among the *Fatp* family members, we observed that only *Fatp1* and *4* are expressed in the control retina, in both the neuroretina and the retinal pigment epithelium. In the neuroretina, *Fatp1* is mostly expressed in photoreceptors. In young adult *Fatp1^−/−^* mice, *Fatp4* expression was unchanged in retinal pigment epithelium and reduced two-fold in the neuroretina as compared to *Fatp1^+/+^* mice. The *Fatp1^−/−^* mice had a preserved retinal structure but a decreased electroretinogram response to light. These mice also displayed a delayed recovery of the b-wave amplitude after bleaching, however, visual cycle speed was unchanged, and both retinal pigment epithelium and photoreceptors presented the same fatty acid pattern compared to controls. In 2 year-old *Fatp1^−/−^* mice, transmission electron microscopy studies showed specific abnormalities in the retinas comprising choroid vascularization anomalies and thickening of the Bruch membrane with material deposits, and sometimes local disorganization of the photoreceptor outer segments. These anomalies lead us to speculate that the absence of *FATP1* accelerates the aging process.

## Introduction

In mammals, the cone and rod photoreceptors (PRs) are responsible for the transformation of light into an electrical signal that is transmitted to the brain. Adjacent to the PRs is the retinal pigment epithelium (RPE), which is essential for nutrition and detoxification of the PR among many other functions [1]. These two tissues are interdependent for their survival and the degeneration of one causes the degeneration of the other, leading to various forms of pigmentary retinopathies and often blindness [1], [2], [3]. PRs comprise a cellular extension called an outer segment containing the visual pigment in membranous disks. These disks are renewed in a cyclical way from the base while the RPE phagocytes the oldest apical disks. If the phagocytosis process is blocked, membranous debris accumulate and PRs degenerate [4]. Due to the permanent renewal of their disks, PRs need a high supply in fatty acids, in particular in docosahexaenoic acid (DHA, 22:6*n*-3), and are mainly dependent on the RPE for this supply [5].

In the rod PRs, the visual pigment rhodopsin is comprised of a protein, opsin, attached to a chromophore, 11*-cis* retinal. When rhodopsin is activated by photon absorption, the retinal molecule is isomerized into all*-trans* retinal and secondarily detached from the protein. The retinoid is then exported toward the RPE. There, a lecithin-retinol-acyl transferase (LRAT) esterifies the retinoid into all*-trans* retinyl ester by addition of a long chain fatty acid (often a palmitate). The resulting retinyl ester is the substrate of RPE65, the RPE isomerase that frees the fatty acid and transforms the retinoid back into 11*-cis* retinol. After oxidation, the formed 11*-cis* retinal is transported back to the PRs to form fresh rhodopsin. This metabolism is called the visual cycle, and many actors participate in this process of which the rate-limiting step is the isomerization catalyzed by RPE65 [1], [6], [7]. Mutations in many of the genes encoding visual cycle proteins alter the visual process, leading to various forms of retinal degenerations or stationary disorders [8], [9], [10]. In addition, the visual cycle generates retinoid by-products such as *N*-retinylidene-*N*-retinylethanolamine (A2E), which interfere with the normal function, in particular lysosomal function, of the RPE. With age, this can lead to the accumulation of lipofuscin, composed of residues generated by incomplete lysosomal digestion in the RPE, which leads to retinal degeneration.

**Table 1:** Sequences of the qPCR primers.

Gene		Primer sequence
*Fatp1*	FR	5′-AGGTCAATGAGGACACGATGGAG-3′ 5′-CTGGTACATTGAGTTAGGGTCCAAC-3′
*Fatp2*	FR	5′-GCCAAGTCTCTGCTGCACTGC-3′ 5′-CTTCAGACCTCCACGACTCCG-3′
*Fatp3*	FR	5′-CCAGAACATAGAGTGGGTCAGACA-3′ 5′-CTTCACTTCCACGATCGTACTGG-3′
*Fatp4*	FR	5′-CTGAAGCTGCCCTGGACCCA-3′ 5′-AGGGCATCCCGCCTAAGGTTG-3′
*Fatp5*	FR	5′-CAAGTTGGGCTGCCCTGTGGC-3′ 5′-GCCCTATCGTATGGTACAGAGG-3′
*Fatp6*	FR	5′-CCAACCTTCGCTTCGATTCCCT-3′ 5′-CATCAGAGGCGAGACTCAGCTT-3′
*Rpe65*	FR	5′-GTGCCACTGCTCATCCACATATTG-3′ 5′-TGCAGGGGAACTGCACAACAACT-3′
*Rhodopsin*	FR	5′-CGCTTCGGGGAGAATCA-3′ 5′-GTAGGGAAGCCAGCAGAT-3′
*Lrat*	FR	5′-GAGCAGCAGTTGGGACTGACT-3′ 5′-TCCCAAGACAGCCGAAGCAAGA-3′
*Rdh5*	FR	5′-TCACCAGTGTCTTGGGCCGCA-3′ 5′-AGGTTGGTCACAGGGGTTCGAA-3′
*Mertk*	FR	5′-CAGTTTTATCCTGATGAGGAAGG-3′ 5′-GAAGGCTGTGTTTCTGGTGAC-3′
*Actin*	FR	5′-CTGGGC CTC GTC ACC CAC ATA-3′ 5′-GACCCAGATCATGTTTGAGACCTT-3′

In order to identify novel regulators of the visual cycle, using two-hybrid experiments, we previously showed that the visual cycle enzymes RPE65 and LRAT physically interact with Fatty Acid Transport Protein 1 (FATP1), an acyl-coA synthase with broad specificity for both long (such as palmitate) and very long chain fatty acids. Systemically, FATP1 is expressed in white adipose tissue, skeletal muscle, heart, brain and skin [11]. FATP1 prevents fatty acid efflux from the cell and as a consequence promotes further transport into the cell. In the retina, the protein is expressed in both RPE and neuroretina (NR) [12]. Our previous studies showed that, *in vitro*, the interaction between FATP1 and RPE65 and LRAT inhibits 11*-cis* retinol. *In vivo*, this inhibitory mechanism could be exploited to regulate the visual cycle, with an increase in FATP1 activity being used to slow the cycle down. This property may decrease the A2E visual cycle byproduct that abnormally accumulates in certain retinal diseases.

**Figure 1:**
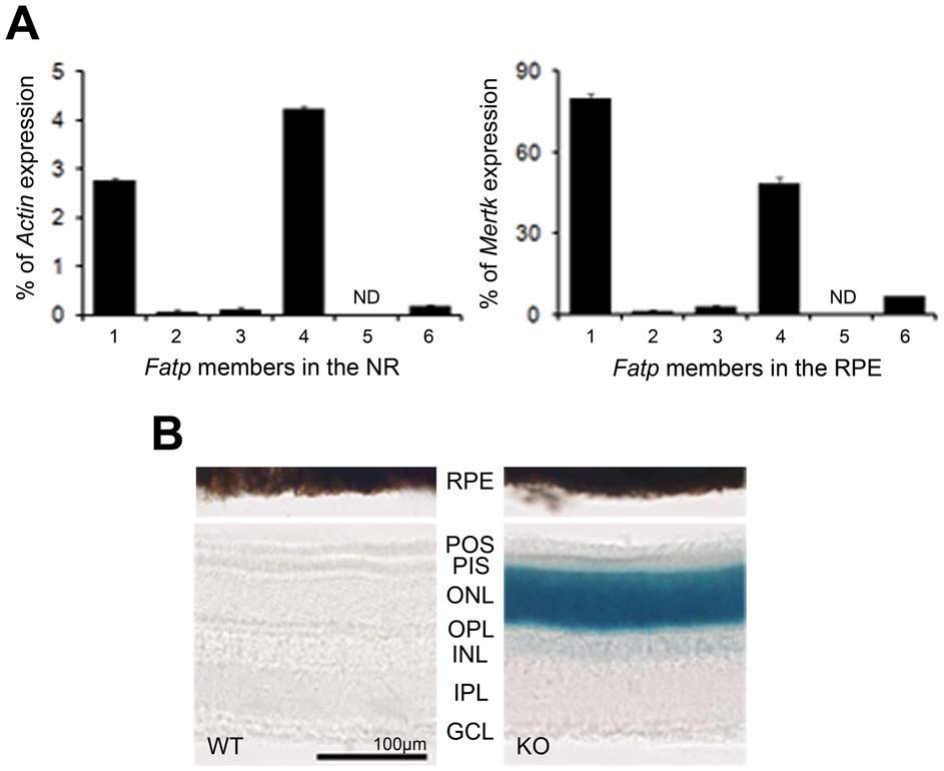
Expression of the Fatp family in the retina. A. Expression of the *Fatp* family in *Fatp1* ^+/+^ neuroretina (NR) and retinal pigment epithelium (RPE). Only *Fatp1* and *Fatp4* were expressed in both tissues. *Fatp4* predominated in the NR and *Fatp1* in the RPE. ND: not detected. Results are expressed as a percentage of *Actin* mRNA expression for NR and as a percentage of *Mertk* mRNA expression for RPE. mRNA was extracted from tissue pools of 12 mice aged 5 months. Bars represent the mean of triplicates ± SEM. B. *Fatp1* expression pattern using the LacZ cassette of the *Fatp1^−/−^* mouse. beta-galactosidase staining of the retinas of *Fatp1* ^+/+^ (WT; without LacZ cassette) and *Fatp1^−/−^* (KO; containing the LacZ cassette) mice. In neuroretina, *Fatp1* is strongly expressed in photoreceptors, and slightly expressed in the inner cells. POS: PR outer segments. PIS: PR inner segments. ONL: outer nuclear layer. OPL: outer plexiform layer. INL: inner nuclear layer. IPL: inner plexiform layer. GCL: ganglion cell layer.

In this study, we used *Fatp1*-deficient mice to test the role of the protein in the visual cycle and on visual function. We found that PR of young *Fatp1^−/−^* mice had a weaker response to light and that recovery of the visual sensitivity after bleaching was slightly delayed. A thorough analysis of the *Fatp1^−/−^* mouse phenotype also showed that old mice had PR outer segment disorganization, irregular Bruch membrane (BM) and abnormal choroid vascularization. These observations suggest that Fatp1 deficiency could induce an accelerated aging of the retina.

**Figure 2:**
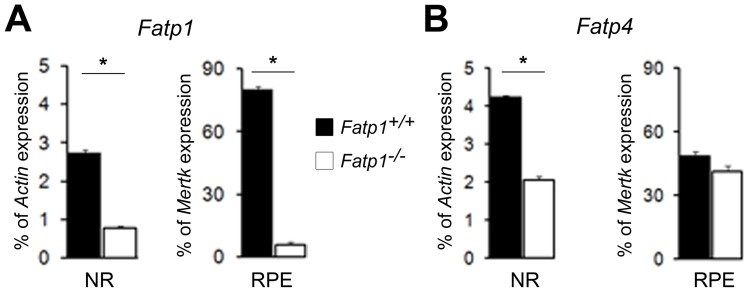
Expression of Fatp1 and Fatp4 in the retinas of *Fatp1* ^+/+^ and *Fatp1* *^−^* ^/*−*^ mice. Expression of *Fatp1* (A) and *Fatp4* (B) in the NR and RPE of *Fatp1* ^+/+^ and *Fatp1^−/−^* mice as determined by qPCR. Results are expressed as a percentage of *Actin* mRNA expression for NR and as a percentage of *Mertk* mRNA expression for RPE. mRNA was extracted from tissue pools of 12 *Fatp1* ^+/+^ and 10 *Fatp1^−^* ^/*−*^ mice aged 5 months old. Bars represent the mean of triplicates ± SEM. * *p*<0.05. *Fatp4* expression is decreased in the *Fatp1^−/−^* NR as compared to wt and expression levels are unchanged in the RPE. There is no compensatory up-regulation of *Fatp4* in the *Fatp1^−/−^* retinas.

## Materials and Methods

### Mice

All animals were handled in strict accordance with the ARVO Statement for the Use of Animals in Ophthalmic Research and with EU directives. Approval from an Ethical Committee is not obligatory in France until 2013. The animal house and the project head must have a permit from the "Direction Départementale des Services Vétérinaires" and all persons handling the mice must be specifically trained. In this work, all protocols were performed under the personal permit number 34–331 (MOP) and the animal house permit number B34–17236. Moreover, all efforts have been made to avoid suffering. *Fatp1^+/−^* mice, generated on a mixed C57BL/6J x 129SvEv genetic background, were kindly provided by Andreas Stahl (Berkeley, California, USA). These mice carried a leucine residue at aa position 450 in Rpe65. They were bred (heterozygous x heterozygous) in the INM animal house in clear plastic cages, subjected to standard light cycles (12 h to 90 lux light, 12 h dark) and were fed *ad libitum* with a standard rodent diet. The genotyping was conducted as previously described [13].

**Figure 3:**
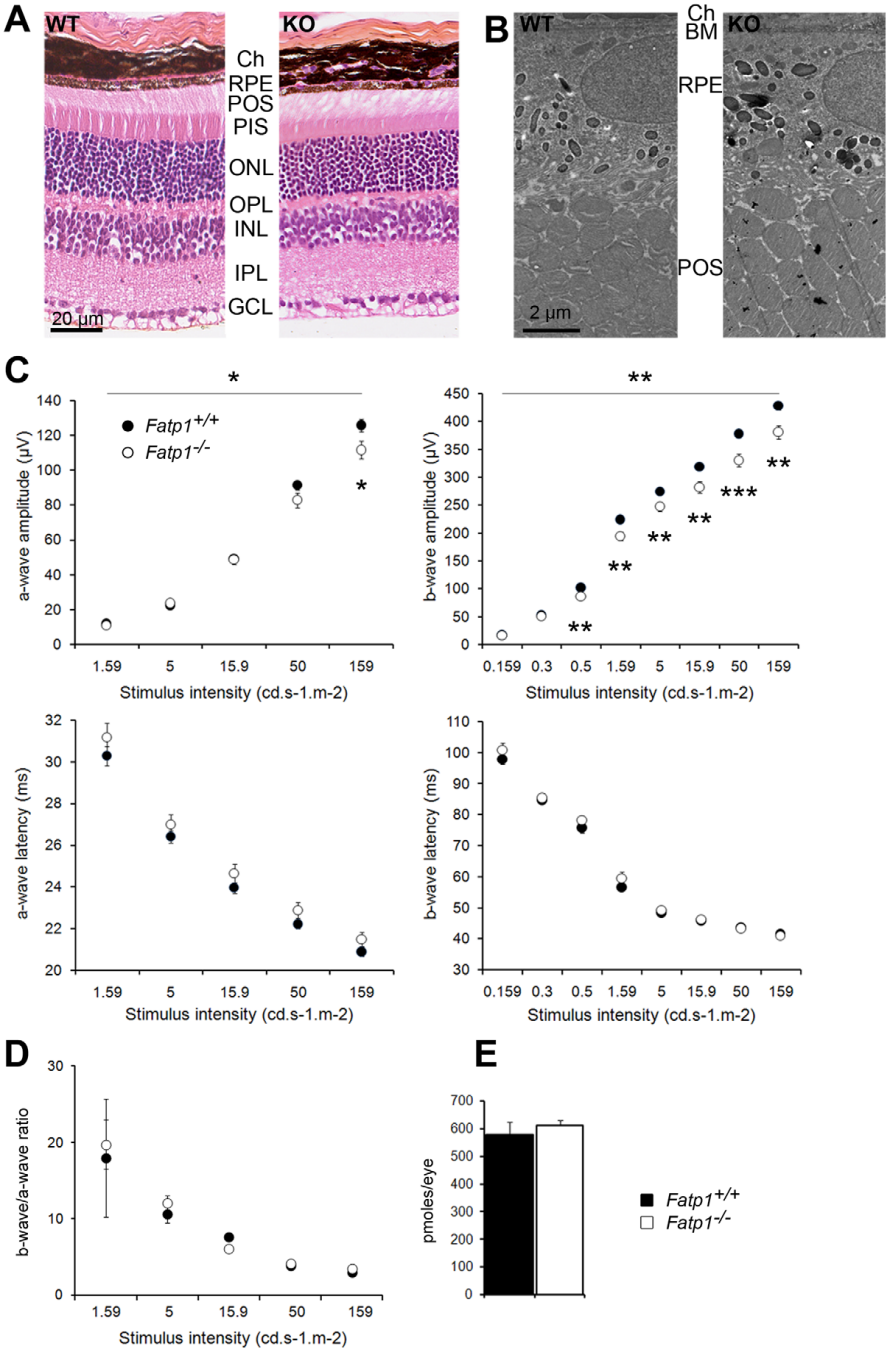
Visual phenotype of the *Fatp1* ^−/−^ mouse. A. Histology of the retina. Ch: choroid. RPE: retinal pigment epithelium. POS: PR outer segments. PIS: PR inner segments. ONL: outer nuclear layer. OPL: outer plexiform layer. INL: inner nuclear layer. IPL: inner plexiform layer. GCL: ganglion cell layer. WT: *Fatp1* ^+/+^. KO: *Fatp1^−/−^*. B. TEM of WT and KO retinas. BM: Bruch membrane. C. Electroretinography of *Fatp1* ^+/+^ (*n* = 29) and *Fatp1^−/−^* (*n* = 23) mice. * *p*<0.05; ** *p*<0.01; *** *p*<0.001. The statistical analyses were performed initially for the whole curve (line above the graph) and subsequently for each point. D. b-wave on a-wave ratio of the ERG experiments. Error bars represent SEM. E. Quantification of the retinal coupled-rhodopsin (*n* = 4 *Fatp1* ^+/+^ and *n* = 4 *Fatp1^−/−^*) in mice aged 5 to 8 months. Error bars represent SEM. The retinal histology is unchanged in the *Fatp1^−/−^* mice. *Fatp1^−/−^* PRs present a lower response to light flashes and this decrease is not due to a decrease in the level of retinal coupled-rhodopsin in these animals.

### Quantitative PCR (qPCR) assay

Five mo-old *Fatp1* ^+/+^ and *Fatp1^−^* ^/*−*^ mice were euthanised by cervical dislocation, the eyes were enucleated and dissected between 9 and 11 a.m. The NR was separated from the RPE-choroid, and then the RPE-choroid was scraped off from the sclera. We chose to scrape off both tissues rather than to enzymatically separate the RPE alone, to minimize the time and the manipulation between the death of the mice and the removal of the tissue. To obtain sufficient quantities of RNA, the tissues from all mice were pooled for each tissue type and total RNA was isolated by the RNeasy mini kit (Qiagen) according to the manufacturer's protocol. Equivalent amounts of total RNA were used for first strand cDNA synthesis using the Verso(TM) cDNA Kit (ThermoScientific). qPCR was performed using Light Cycler FastStart DNA MasterPLUS SYBR Green I kit (Roche). Amplifications from NR were normalized to actin levels, and those from RPE-choroid were normalized to the RPE-specific gene *Mertk*, in order to avoid an incorrect estimation due to the presence of choroid RNA. Primers used for amplification are summarized in Table 1.

**Figure 4:**
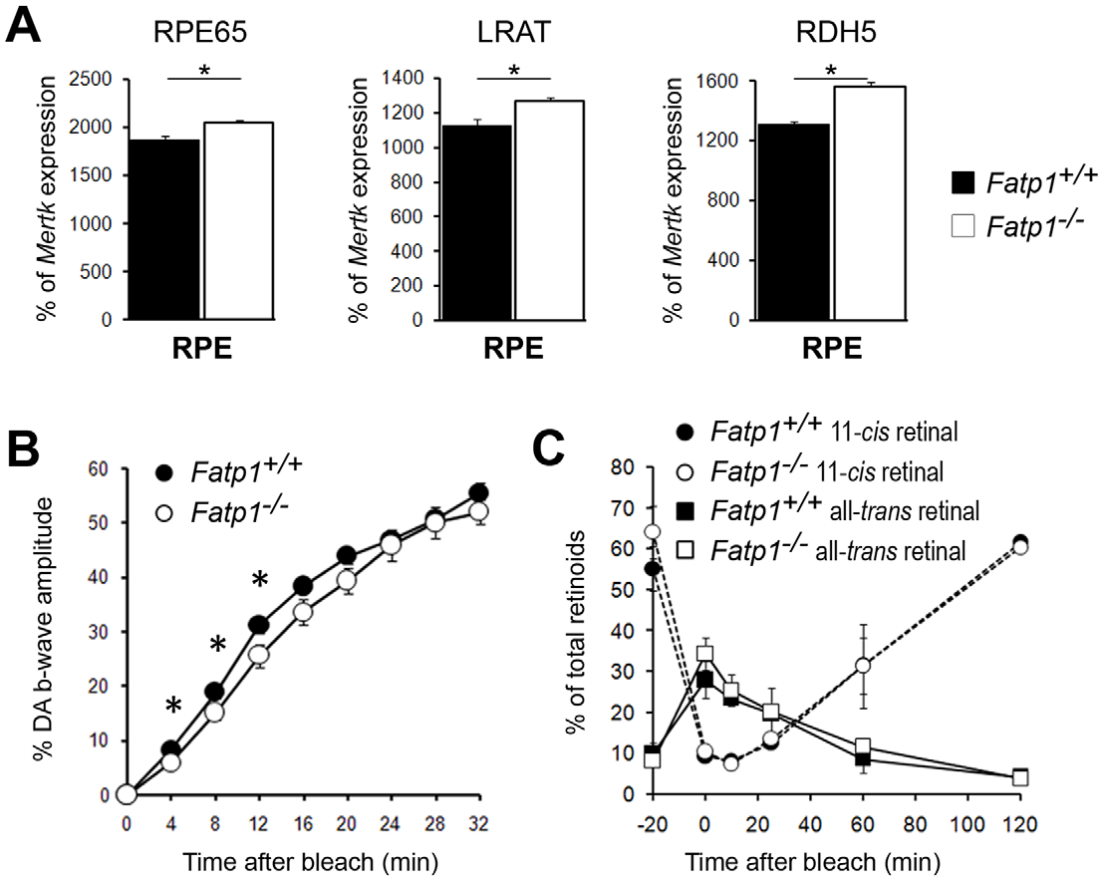
The visual cycle in 5 to 8 mo-old *Fatp1* ^−/−^ mice. A. Expression levels of the main visual cycle genes, Rpe65, Lrat and Rdh5. Bars represent the mean of triplicates ± SEM. * *p*<0.05. The genes were slightly deregulated with a 9.6% increase observed for *Rpe65*, a 12.5% increase for *Lrat* and a 19.5% increase for *Rdh5*. B. Recovery of the b-wave amplitude after bleaching in 5 to 8 mo-old mice (*n* = 29 *Fatp1* ^+/+^ and *n* = 21 *Fatp1^−/−^*). Error bars represent SEM. * *p*<0.05. C. Quantification of the 11-*cis* retinal and all-*trans* retinal levels in the whole eye. Error bars represent SEM. The recovery of the electroretinogram is delayed in *Fatp1^−/−^* mice but this difference is not linked to a visual cycle kinetic defect.

### Histology

All animals were sacrificed by vertebral dislocation. Eyes were rapidly enucleated and fixed in 4% PFA, 24 h at 4°C. Eye cups were i) embedded in paraffin and cut into 5 µm sagittal sections, ii) embedded in 5% agarose and cut into 50 µm sagittal sections, or iii) soaked in 40% sucrose, embedded in freezing medium (OCT) and cut into 10 µm sagittal sections. For HES coloration, sections were deparaffined, labeled with hematoxilin/eosin/safran, rinsed and mounted in Moeviol. For betagalactosidase staining, agarose sections were transferred into the freshly prepared staining solution (200 mM MgCl2, 500 mM K4Fe(CN)6, 500 mM K3Fe(CN)6, 4% X-gal, 2% NP-40, in 1X PBS), kept in the dark and incubated at 30°C for 1 hour. Staining was stopped by several washing in PBS. Oil red O coloration was performed as previously described [14]. Eye sections with demonstrated lipid accumulation were used as positive controls and kindly provided by Alexandra Provost (CERTO, Paris, France) [14].

**Figure 5:**
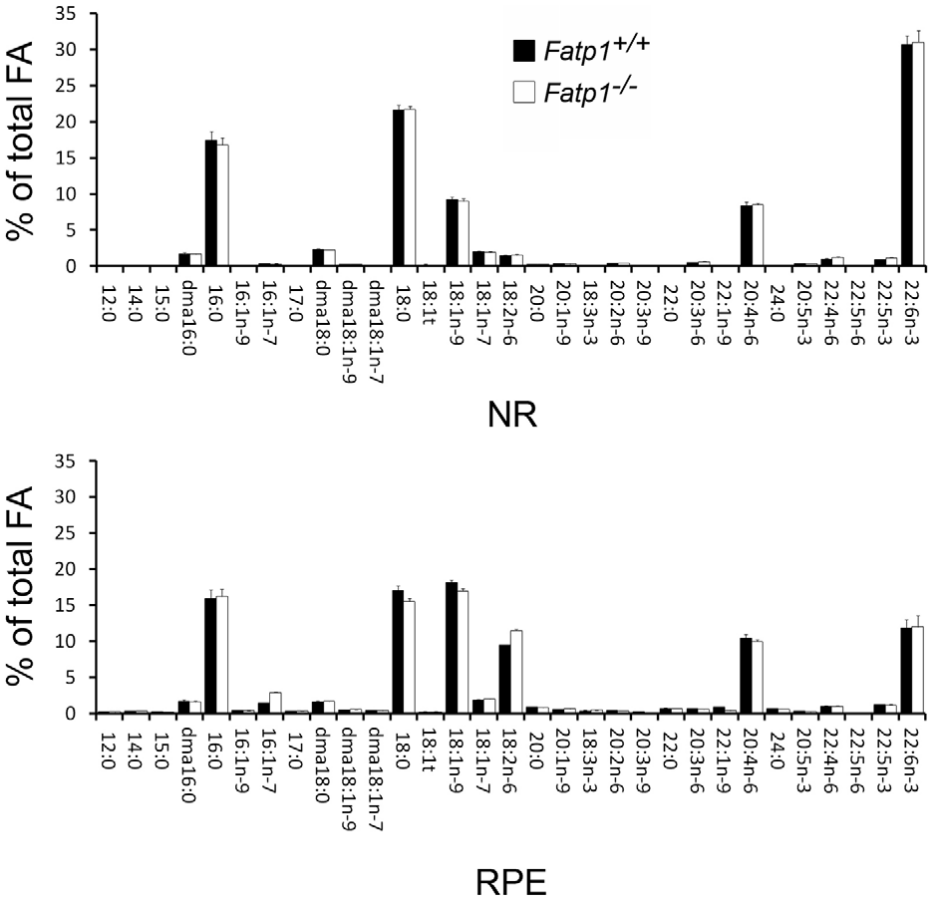
Fatty acid composition in the NR and RPE in 8 mo-old *Fatp1* ^+/+^ and *Fatp1^−/−^* mice. *Fatp1* ^+/+^ (n = 5) and *Fatp1^−/−^* (n = 7). 16:0: palmitate. 22:6n-3: DHA. The functional visual difference between *Fatp1^−/−^* and *Fatp1* ^+/+^ mice is not due to a different fatty acid composition.

### Transmission electron microscopy (TEM)

Eyes were rapidly enucleated and the corneas were removed prior to immersion in a solution of 3.5% glutaraldehyde in Sorensen's buffer (0.1 M, pH 7.4) overnight at 4°C. The tissues were then rinsed in Sorensen's buffer and post-fixed in a 1% osmic acid for 2 h in the dark at room temperature. After two rinses, the tissues were dehydrated in a graded series of ethanol solutions (30–100%) and embedded in EmBed 812 using an Automated Microwave Tissue Processor for Electronic Microscopy, Leica EM AMW. All samples were processed the same way: a part of the eye was cut in a triangle with its summit at the optic nerve and the base at the periphery. Cutting was always initiated from the summit. The sections observed were thus all near the optic nerve. Sections (60 nm thickness; Leica-Reichert Ultracut E) were counterstained with uranyl acetate and observed using a Hitachi 7100 TEM (Centre de Ressources en Imagerie Cellulaire de Montpellier, France). This study was not quantitative as we observed few sections from one part of each eye. However, we evaluated the thickness of the Bruch's membrane by measuring both the thickest and the thinnest parts in 6 fields throughout the retinal section. We then calculated the median value for each eye.

**Table 2:** Abnormalities observed in aged mice by TEM.

	*Fatp1* ^+/+^ (*n* = 9)	*Fatp1^−/−^* (*n* = 15)
Abnormal choroidal vessels	0/9	6/15 with only large vessels 2/15 with no vessels 4/15 with continuous vascular layer
Abnormal Bruch membrane	0/9	10/15
Disorganized PR outer segments	0/9	6/15

**Figure 6:**
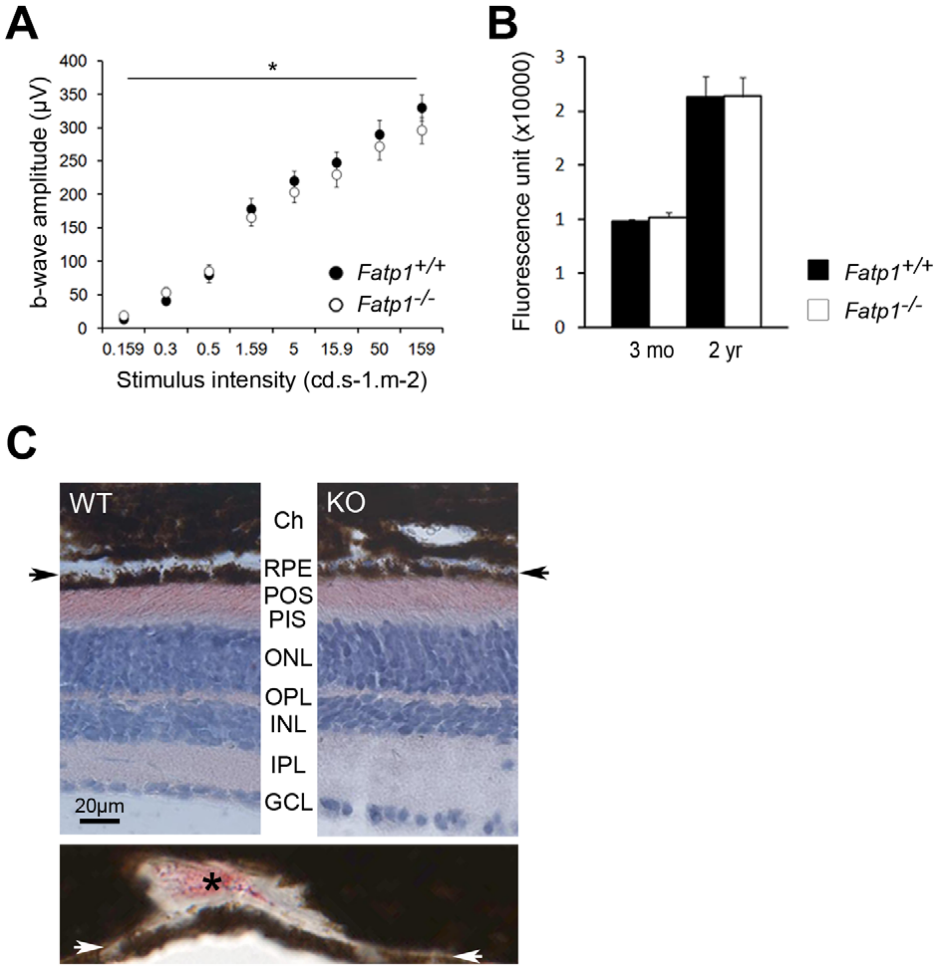
Aging of the *Fatp1* *^−/−^* retina. A. Electroretinography of *Fatp1* ^+/+^ (*n* = 5) and *Fatp1^−/−^* (*n* = 11) mice, * *p* = 0.021 for the whole curve. B. Quantification of autofluorescence in young (*n* = 3 *Fatp1* ^+/+^ and *n* = 4 *Fatp1^−/−^*) and old (*n* = 6 *Fatp1* ^+/+^ and *n* = 5 *Fatp1^−/−^*) mice. The values are expressed in arbitrary fluorescent units from the machine. For each mouse, the result is obtained by summing up all values between 520 and 640 nm taken every 10 nm. Error bars represent SEM. *Fatp1^−/−^* PRs remains less responsive to light with age. *Fatp1^−/−^* mice do not accumulate more autofluorescence, thus presumably more lipofuscin, than *Fatp1* ^+/+^ with age. C. Upper panel: neutral lipid labeling of *Fatp1* ^+/+^ (WT) and *Fatp1^−/−^* (KO) retinas. Ch: choroid. RPE: retinal pigment epithelium. POS: PR outer segments. PIS: PR inner segments. ONL: outer nuclear layer. OPL: outer plexiform layer. INL: inner nuclear layer. IPL: inner plexiform layer. GCL: ganglion cell layer. Arrows indicate the BM. Lower panel: positive control at the same magnification (see Material and methods). Arrows indicate the BM as in the upper panel. The asterisk indicates positive ORO labeling. *Fatp1^−/−^* or *Fatp1* ^+/+^ mice showed no Oil red O labeling in the BM.

**Figure 7:**
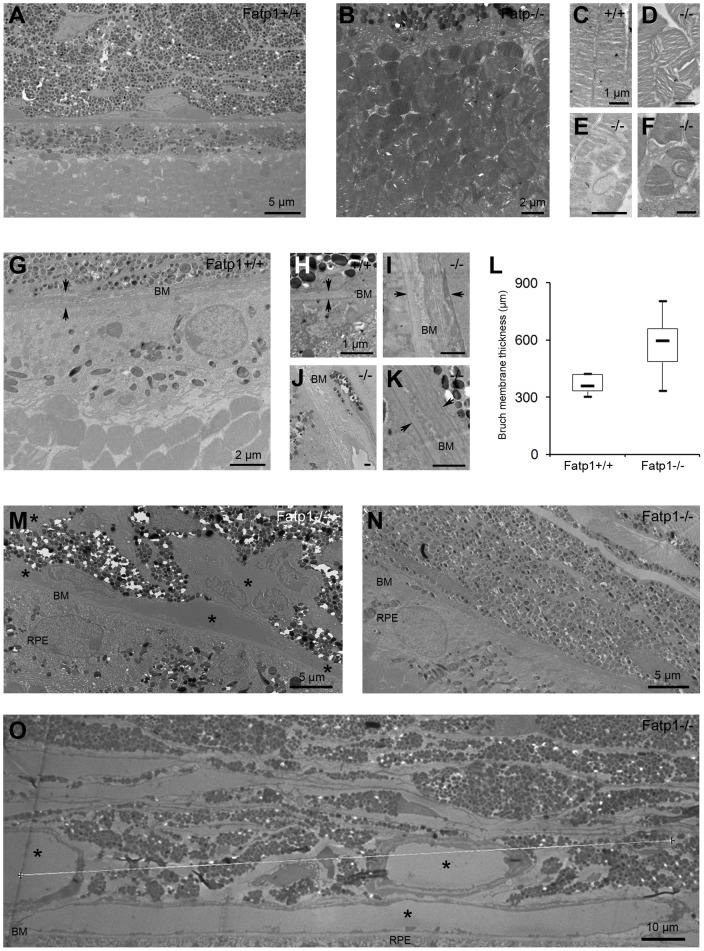
Ultrastructural aging of the *Fatp1* *^−/−^* retina. A: TEM at low magnification of a *Fatp1* ^+/+^ retina. PR outer segments in *Fatp1^−/−^* at low magnification (B), in *Fatp1* ^+/+^ at high magnification (C), in *Fatp1^−/−^* mice at high magnification (D–F). Certain *Fatp1^−/−^* mice had PR outer segment disorganization. G: *Fatp1^−/−^* retina at low magnification showing a thick BM. H: *Fatp1* ^+/+^ BM at high magnification. I-K: *Fatp1^−/−^* retina at high magnification showing a thicker BM than age-matched control. L: Box plot graph showing the thickness of the BM between *Fatp1* ^+/+^ and *Fatp1^−/−^* retinas (solid line indicates the median; *p*<0.05). M-O: *Fatp1^−/−^* retinas at low magnification showing numerous vessels along the BM (M), no vessels (N) and large vessels (O). Arrows indicate the thickness of the BM. Asterisks indicate vessels. The majority of *Fatp1^−/−^* mice exhibited an irregular or laminar thicker BM. Most of the *Fatp1^−/−^* mice showed strikingly modified choroidal vessels, ranging from presence of only large vessels to absence of vessels.

### Electrophysiology

All electrophysiological examinations were conducted using the Visiosystem (SIEM, France). Animals were prepared and electroretinograms (ERGs) were performed with cotton electrodes as previously described [15]. For adaptation-ERG, the mice were subjected to seven repetitions of a 1.59 cd.s^−1^.m^−2^ blue flash, the seven b-wave amplitudes were averaged and considered as dark-adapted control. The mice were then bleached 2 min at 300 lux, placed again in the dark and subjected to the same series of flashes to verify the abolition of the b-wave. This series of flashes was repeated every 4 min from 0 to 32 min, to observe the recovery of the b-wave.

### Rhodopsin quantification

All manipulations were performed under dim red light. Immediately after cervical dislocation of the mice, eyes were enucleated, and homogenized in 1 ml of PBS, pH 7.2 and centrifuged at 140,000 g, 4°C for 20 min. The pellet was re-suspended in 1 ml of PBS pH 7.2 with 1% Triton X-100 and then incubated at 4°C for 1 h. The homogenate was centrifuged at 140,000 g, 4°C for 20 min. Ten µl of hydroxylamine was added to the supernatant to achieve a final concentration of 20 mM, and absorption spectra of the samples were obtained before and after complete bleaching with a 60-W incandescent bulb. The concentration of rhodopsin was determined by the decrease in absorption at 500 nm and the molar extinction of rhodopsin (e = 42,000 liter/mol/cm).

### Retinoid quantification

Pupillary dilatation was obtained with one instillation of 0.5% tropicamide (Mydriaticum, Théa, France) in each eye. Animals were sacrificed by cervical dislocation before bleaching (5000 lux, 20 min) or at different times thereafter (0, 5, 15, 30, 60 and 120 min). The eyes were rapidly enucleated, frozen in liquid nitrogen and conserved at −80°C until use. The extraction was performed as described [16] with a few modifications: the eyes were homogenized in 800 µl of 3 M formaldehyde; after incubation, 1.5 ml of dichloromethane was added; the volume of hexane added was 3 ml; the extracts were dissolved at the end in 20 µl of ethanol for HPLC. The analyses were performed with a Varian HPLC system equipped with a C18 Isis column (4.6×250 mm) (Macherey-Nagel) and a Prostar 330 diode array detector. The retinoids were quantified from the peak areas using calibration curves determined with established standards.

### Fatty acid profile analysis of the retina

At the age of 4 and 8 months, the mice were euthanised, the eyes enucleated, and the NR removed. RPE-choroid was scraped and collected in 1X PBS, then centrifuged to remove the PBS. Tissues were stored dry at −80°C. Lipids were extracted [17] and fatty acids were transmethylated [18]. Fatty acid methyl esters were analyzed by gas chromatograph (5890 series II; Hewlett-Packard, Palo Alto, CA) equipped with a split/splitless injector, a flame-ionization detector, and a CPSil88-silica capillary column (100 m×0.25 mm ID, film thickness 0.20 μm; Varian). The injector and the detector were maintained at 250°C and 280°C, respectively. Hydrogen was used as a carrier gas (inlet pressure, 210 kPa). The oven temperature was fixed at 60°C for 1 minute, increased to 85°C at a rate of 3°C/min and then to 190°C at a rate of 20°C/min and left at this temperature for 65 minutes. Fatty acid methyl esters were identified by comparison with commercial standards.

### Autofluorescence measurements

For each mouse, one eye was homogenized two times 10 minutes in 1X PBS buffer without MgCl_2_ using bead-filled tubes (MagNA Lyser, Roche). Tubes were centrifuged 10 minutes at 100 g to separate the scleral debris. The fluorescence in the supernatant was measured using a Safire II micro plate reader with an excitation of 405 nm and an emission of 520 to 640 nm. The values are expressed in arbitrary fluorescent units from the machine. For each mouse, the result is obtained by summing up all values between 520 and 640 nm taken every 10 nm.

### Statistics

All data, except ERGs, were analyzed using the non parametric Mann & Whitney test due to the sample size, with a significant threshold level set at ≤5%. For ERGs, due to the complexity of the data, a Hotelling T-square test was performed to assess the overall difference between each pair of mean vectors from the longitudinal data. In those cases where significance was ≤5%, a Welch 2 sample T-test, corrected for multiple comparisons by the Bonferroni method, was performed at each time point to assess the difference between the two means. All computations where performed using the R package (http://www.r-project/org) [19].

## Results

### *Fatp* expression in the retina

We first examined the expression of all *Fatp* members in the RPE and NR of *Fatp1* ^+/+^ mice by qPCR. Only *Fatp1* and *Fatp4* were expressed in both tissues, with high levels detected in the RPE relative to *Mertk*, and in the NR, relative to *Actin* mRNA. *Fatp4* predominated in the NR and *Fatp1* in the RPE (Figure 1A). To precisely determine the cells expressing *Fatp1* among the neuroretinal cells, we took advantage of the fact that Fatp1*^−^* ^/*−*^ mice were generated by replacing the first coding exon of *Fatp1*, which encodes amino acids 1 to 56, with a targeting cassette containing an nlsLacZ sequence [13]. We thus studied *Fatp1* expression with a beta-galactosidase staining. Due to pigmentation, it was not possible to observe the labeling in RPE. In NR, the strongest labeling was observed in the PR cells (Figure 1B). The inner nuclear layer (INL) and ganglion cell layer (GCL) nuclei were also slightly labeled.

In Fatp1*^−^* ^/*−*^ mice, Kim *et* *al*. [13] previously verified that there was no compensatory up-regulation of other members of the *Fatp* family (*Fatp2* to *5,* as *Fatp6* was not yet discovered) in skeletal muscle and white adipose tissue, the two major sites of *Fatp1* expression known at this time. We analyzed the expression of the 6 *Fatp* family members in the *Fatp1^−/−^* retina. Accordingly, *Fatp1* expression was effectively at background levels in *Fatp1^−/−^* eyes (Figure 2A) and *Fatp4* was the only member expressed consistent with our results in wt retina. However, *Fatp4* expression was decreased in the *Fatp1^−/−^* NR (2-fold) as compared to wt whereas expression levels were unchanged in the RPE (Figure 2B). *Fatp4* is the closest member of the family to *Fatp1* but does not seem to compensate the absence of *Fatp1* at the mRNA level.

### Visual phenotype of young *Fatp1^−^* ^/*−*^ mice

We studied the visual phenotype of young 5 to 8 mo-old adult mice. The retinal histology of *Fatp1^−/−^* was similar to that of wt mice (Figure 3A) and we observed no differences with TEM (Figure 3B). PRs appeared similar in morphology and were present in the same number in both genotypes. We then assessed the functionality of the wt and *Fatp1^−/−^* retinas by ERG recordings. The light stimulus increased gradually from an only-rod stimulation to a mixed rod-cone stimulation. Latencies were similar in wt and *Fatp1^−/−^* mice. In contrast, the amplitudes of the ERG responses were weaker in *Fatp1^−/−^* mice than in wt, with the amplitude differences *Fatp1^−/−^ vs Fatp1^+/+^* being more pronounced for the highest light intensities (maximum b-wave amplitude  = 88.9% of the wt; Figure 3C). This difference appears to be due to a dysfunction of the PRs (a-wave) and not of the interneurons (b-wave) as the ratio of b-wave over a-wave was not decreased in *Fatp1^−/−^* mice (Figure 3D). It was not related to a decrease in photon catch by *Fatp1^−/−^* PRs as active rhodopsin levels were the same in both genotypes (Figure 3E).

To examine whether the decrease in ERG responses could be related to a dysfunction of the visual cycle, we first verified whether the absence of *Fatp1* deregulated the expression of three of the main genes involved in the visual cycle, i.e. *Rpe65*, *Lrat* and *Rdh5* (Figure 4A). In the absence of *Fatp1*, the expression of the 3 genes was only slightly (but significantly) increased by 9.6%, 12.5% and 19.5% respectively. We then studied the effect of the absence of Fatp1 on the recovery of the b-wave after bleaching. In the *Fatp1^−/−^* mice, we observed a delay in the first minutes of recovery followed by normalization at 16 min (Figure 4B). This could be explained by a delay in the regeneration of the chromophore 11*-cis* retinal in the RPE. Therefore, we measured this regeneration directly by measuring the level of each retinoid before and after bleaching during re-adaptation in the dark and observed no differences between *Fatp1* ^+/+^ and *Fatp1^−/−^* retinal extracts at any timepoint (Figure 4C). These results suggested that the lack of *Fatp1* does not have a strong impact on chromophore regeneration. Thus, the difference in b-wave recovery observed *in vivo* between wt and *Fatp1^−/−^*mice did not seem to be due to a major deregulation of the visual cycle.

### Fatty acid composition in the retina of *Fatp1^−/−^* mice

We observed an approximately 10% decrease in the ERG amplitudes and a delayed ERG b-wave amplitude recovery after saturating flash in *Fatp1^−/−^* mice as compared to wt. These abnormalities were reminiscent of observations made on n-3 fatty acid-deficient animals. The fatty acid docosahexaenoic acid (DHA, 22:6*n*-3) is highly enriched in membrane phospholipids of the brain and retina, and in particular PR outer segments. Reduced ERG amplitudes have been reported in animals raised in DHA precursor-deficient diets, and a delayed recovery of ERG b-wave amplitude after a b-wave saturating flash has been observed [20], [21], [22]. This could be explained by reduced rhodopsin efficiency due to lower protein diffusion in the membrane of the discs. As *Fatp1* is absent and *Fatp4* less expressed in the NR of *Fatp1^−/−^* mice, it was possible that the lipid composition of the PR outer segments was abnormal, leading to photoreceptor dysfunction. We therefore analyzed the fatty acid composition of the *Fatp1^−/−^* and *Fatp1* ^+/+^ NR and RPE for 32 fatty acids at the age of 4 and 8 months. No changes of the fatty acid composition were seen in the neural retina, which include PRs, nor in the RPE (Figure 5).

### Histological anomalies in the retina of old *Fatp1^−/−^* mice

We analyzed the retinal structure and function of 22 to 26 mo-old wt and *Fatp1^−/−^* mice. Both genotypes mice had lower ERG responses compared to young animals (Figure 3C). However, responses of older *Fatp1^−/−^* mice remained lower than that of age-matched controls (maximum b-wave amplitude  = 89.8% of wt; Figure 6A). We found no evidence of abnormal dark adaptation of the *Fatp1^−/−^* mice after bleaching, but this was possibly due to the small number of old animals available for this protocol (3 *Fatp1^+/+^* and 4 *Fatp1^−/−^*). We assayed for the presence of lipofuscin, an autofluorescent byproduct of the visual cycle that accumulates with age and is often observed in age-related macular degeneration (AMD) retina. However, there was no difference in the amount of autofluorescence between wt and *Fatp1^−/−^* animals (Figure 6B). As age-dependent lipid accumulation has been previously described in Bruch membrane (BM) [14], [23], we searched for lipid droplet deposition in aged mice. However, *Fatp1^−/−^* or wt mice showed no Oil red O labeling in the BM (Figure 6C). We also studied the retinal structure by TEM. In both *Fatp1^+/+^* (Figure 7A) and *Fatp1^−/−^* mice, we observed classical hallmarks of retinal aging including a decrease in the number of rows of PR nuclei (varying from 5 to 10 rows, mean of 7.7 in *Fatp1^+/+^* and 8 in *Fatp1^−/−^*), thickening of the BM, RPE thinning, enlarged or absent RPE basal infoldings and accumulation of lipofuscin granules. Furthermore, in addition to these common signs of aging, some *Fatp1^−/−^* mice (6/15) had PR outer segment disorganization (Figure 7B–F). In the sections analyzed, there were also greater variations in BM thickness in *Fatp1^−/−^* compared to *Fatp1^+/+^* mice (*p*<0.05; Figure 7L). Two thirds of the *Fatp1^−/−^* mice (10/15) exhibited an irregular BM or a BM containing laminar deposits (Figures 7A and 7G-K). Most of the *Fatp1^−/−^* mice (12/15) showed strikingly modified choroidal vessels. In some cases (6/15), only large vessels were observed suggesting that small vessels had disappeared and in two mice (2/15), there were even no detectable vessels in the sections analyzed (Figure 7M–O). Yet, in other mice (4/15), choroidal vessels had a normal size but were more numerous, forming a continuous layer under the BM. All *Fatp1^−/−^* mice examined by TEM exhibited at least one of these particular additional abnormalities (Table 2).

## Discussion

We previously demonstrated that FATP1 interacts *in vitro* with RPE65 and inhibits the isomerase activity [12]. As a follow up to this work, we analyzed the expression of every member of the *Fatp* gene family in the retina, and we studied *Fatp1^−/−^* mice of various ages to test the effect of Fatp1 deficiency on the visual phenotype and the visual cycle *in vivo*.

We demonstrated that only *Fatp1* and *4* are expressed in NR and RPE, and that, in NR, *Fatp1* is mostly expressed in the PR. Similarly, *Fatp1* and *4* are the only members of the *Fatp* family expressed in adipose tissue and skeletal muscle, two tissues with a high lipid metabolism [11]. This similarity is not surprising as the RPE is the major lipid provider for PRs, which need an important supply of lipids to enable the continuous renewal of their outer segment membranous disks. It has been shown that in skeletal muscle and adipose cells (but not in the heart [24]), Fatp1 translocates to the plasma membrane during postprandial periods and is responsible for the insulin-induced long chain fatty acid uptake. In contrast, Fatp4 participates in basal uptake [25]. In the RPE, we show that *Fatp1* is more prominent than *Fatp4*. This is consistent with the fact that the RPE is in contact with the choroidal blood supply and thus with highly changing concentrations of fatty acids. In contrast, PRs receive their fatty acids from the RPE, probably in more regulated basal levels, thus explaining the higher levels of *Fatp4* as compared to *Fatp1* in the NR.

In Fatp1-deficient mice, *Fatp4* expression is not increased in the retina as compared to controls. This is also the case for muscle and adipose tissues [13] indicating that *Fatp4* expression does not compensate for the lack of *Fatp1* expression. Moreover, *Fatp4* expression was, surprisingly lower in the *Fatp1^−/−^* NR compared to controls. A previous study on a pair of twins (1 obese, 1 lean) reported that Fatp4 expression is up-regulated by environmental factors such as, in this case, acquired obesity [26]. Therefore, *Fatp4* could be less expressed in the *Fatp1^−/−^* NR, because the supply in fatty acid is reduced from the RPE, due to the absence of Fatp1, so that less Fatp4 is needed to facilitate the fatty acid entrance into the PR. However, we showed that this reduction had no significant effect on the lipid composition of the NR at 4 and 8 months of age.

Although FATP1 has been previously described as an actor of the visual cycle *in vitro* [12], in the present study, we showed that the Fatp1-deficient mice do not display a decreased formation of 11*-cis* retinal after bleaching. This could be explained by compensation between Fatp1 and Fatp4 proteins, which are 60% homologous. Phylogenetic analyses reveals that *Fatp1* and *Fatp4* are both the orthologs of a single Drosophila *fatp* gene, suggesting a close function for Fatp1 and Fatp4 [27]. Interestingly, fatp deficiency causes the loss of photoreceptors by a caspase-dependent death, suggesting an important role for fatp in PR survival [28]. Transcomplementation analysis to rescue PR degeneration in *Drosophila fatp* mutants by expressing mouse Fatp1 or Fatp4 genes would be necessary to address the functional relationship of Fatp1 and/or Fatp4 with fatp.

We observed a decrease in the ERG amplitudes and a slower recovery of the b-wave amplitude after bleach in the *Fatp1* ^−/−^ mice. We hypothesized that these ERG anomalies were due to disequilibrium in the fatty acid composition. However, fatty acids were not differentially distributed in NR and RPE of *Fatp1^−/−^* and *Fatp1* ^+/+^, suggesting that in the absence of Fatp1, the fatty acid supply remains sufficient. In addition, as *Fatp1^−/−^* mice fed a normal diet do not exhibit anomalies in intramuscular fatty acid metabolites, insulin sensitivity, body composition (lean and fat mass) or plasma parameters (glucose, insulin, fatty acids, triacylglycerol) [13], it is likely that these differences were due to a specific effect on the eye. One possibility is that the absence of Fatp1 could modify the mitochondrial metabolism. It was previously shown that a part of the cell's Fatp1 content localizes to the outer mitochondrial membrane and that its overexpression in muscle increases the glucose uptake and the PDH activity [29], [30]. Moreover, Fatp1 directs fatty acids towards oxidation, and not storage, in skeletal muscle, liver and adipose tissue [31], [32], [33], all tissues with a high lipid metabolism as in the retina. Fatp1 also directly increases the fatty acid transport in the mitochondria, where it interacts physically with the carnitine palmitoyltransferase 1 (CPT1) [30], the enzyme that passes the co-A fatty acids towards both membranes of the mitochondria. The absence of Fatp1 could therefore decrease the efficacy of the fatty acid oxidation by the mitochondria and thus the production of energy. As the retina is highly in demand of energy for phototransduction, ionic transport and metabolism, a moderate decrease in its mitochondrial supply could result in functional modifications as observed in the *Fatp1^−/−^* mice.

Aging retinas of Fatp1-deficient mice showed strikingly abnormal features, with some individual variations that could be explained by the mixed C57BL/6J ×129SvEv genetic background. There were abnormal depositions of membranous materials into the BM, with a rather diffuse accumulation within and along the BM. This is reminiscent of basal laminar deposits observed in human age-related macular degeneration (AMD) [34]. Similar deposits are also observed in mouse models of AMD fed with high fat diet such as *ApoE^−/−^*, *CD36^−/−^*, *SR-BI^−/−^*, *LDLR^−/−^* mice [14], [35], [36], [37], [38], [39]. In *LDLR^−/−^* and *SR-BI^−/−^* mouse models, lipid droplets were found in BM that are labeled with oil Red O [14], [23]. We did not observe such droplets (nor oil Red O labeling) but more diffuse accumulation within and along the BM, the composition of which remains to be elucidated.

In addition to BM deposits, *Fatp1^−/−^* mice had disorganization of PR and abnormal choroidal vessels. In humans, it is believed that extracellular deposits in BM lead to reduced nutriment transport and to secondary injury of the RPE, choroid and PR. Indeed, the majority of the Fatp1-deficient mice (12/15) presented an abnormal choroidal vascularization. Many of them had only large vessels or no vessels at all. In both cases, the capillaries of the choroid had disappeared, an observation that was also reported in the *CD36^−/−^* model of AMD [36]. Conversely, a smaller number of mice featured an almost continuous layer of capillaris under the BM. This could represent a first step towards breaking of the BM and neovascularization into the subretinal space, as occurs in wet AMD. In contrast to previously described AMD mouse models involving lipid metabolism such as *ApoE^−/−^*, *CD36^−/−^*, *SR-BI^−/−^*, *LDLR^−/−^* mice [14], [35], [36], [37], [38], [39], Fatp1-deficient mice did not show drusen and oil red O labeling or abnormal lipofuscin accumulation. However, it should be stressed that these mouse models require high fat diet (often used to mimic the human alimentation in industrialized countries) for several months in order to display these anomalies [14], [37], [38]. Additional investigations with Fatp1-deficient mice subjected to high fat diet would be necessary to examine whether or not they develop these abnormalities.

In conclusion, we showed that Fatp1-deficient mice exhibit accelerated aging of the outer retina, and that these features were not linked to abnormal visual cycle kinetics or to lipid composition of photoreceptor and RPE.
